# Postmitotic Specification of *Drosophila* Insulinergic Neurons from Pioneer Neurons

**DOI:** 10.1371/journal.pbio.0060058

**Published:** 2008-03-11

**Authors:** Irene Miguel-Aliaga, Stefan Thor, Alex P Gould

**Affiliations:** 1 Division of Developmental Neurobiology, Medical Research Council National Institute for Medical Research, London, United Kingdom; 2 Department of Clinical and Experimental Medicine, Linkoping University Medical School, Linkoping, Sweden; Stanford University, United States of America

## Abstract

Insulin and related peptides play important and conserved functions in growth and metabolism. Although *Drosophila* has proved useful for the genetic analysis of insulin functions, little is known about the transcription factors and cell lineages involved in insulin production. Within the embryonic central nervous system, the MP2 neuroblast divides once to generate a dMP2 neuron that initially functions as a pioneer, guiding the axons of other later-born embryonic neurons. Later during development, dMP2 neurons in anterior segments undergo apoptosis but their posterior counterparts persist. We show here that surviving posterior dMP2 neurons no longer function in axonal scaffolding but differentiate into neuroendocrine cells that express insulin-like peptide 7 (Ilp7) and innervate the hindgut. We find that the postmitotic transition from pioneer to insulin-producing neuron is a multistep process requiring retrograde bone morphogenetic protein (BMP) signalling and four transcription factors: Abdominal-B, Hb9, Fork Head, and Dimmed. These five inputs contribute in a partially overlapping manner to combinatorial codes for dMP2 apoptosis, survival, and insulinergic differentiation. Ectopic reconstitution of this code is sufficient to activate Ilp7 expression in other postmitotic neurons. These studies reveal striking similarities between the transcription factors regulating insulin expression in insect neurons and mammalian pancreatic β-cells.

## Author Summary

Genetic studies using invertebrate model organisms such as *Drosophila* have provided many new insights into the functions of insulin and related peptides. It has, however, been more difficult to use *Drosophila* to study the regulation of insulin, at least in part because the relevant insulinergic cell lineages were not well characterised. Here, we have identified a cell lineage that generates a single *Drosophila* insulin-producing neuron. This neuron first functions as a pioneer, guiding the axons of other neurons within the central nervous system of the embryo. It then develops long axons that exit the central nervous system to innervate the gut and also begins to express an insulin-like peptide. Genetic analysis identifies four transcription factors and one extrinsic signal that instruct the pioneer neuron to become an insulin-producing neuron. The analysis also reveals similarities between the genetic programmes specifying insulin production by *Drosophila* neurons and mammalian pancreatic ß-cells. This suggests that *Drosophila* may, in the future, prove a useful model system for identifying new regulators of human insulin production.

## Abstract 3

A genetic analysis in the fruit fly reveals similarities between the transcriptional programmes regulating insulin production in mammalian pancreatic β-cells and insect neurons.

## Introduction

Insulin, a key regulator of carbohydrate and lipid metabolism, is secreted by the ß-cells of the pancreas [1]. Deficiencies in insulin signalling underlie type I diabetes and also the more widespread type II diabetes often associated with obesity [2,3]. Considerable effort, therefore, has been focused on understanding the development and differentiation of ß-cells [4–6], resulting in the identification of a number of mammalian regulators of insulinergic identity. However, the precise lineage relationships between progenitor cells and each of the five different postmitotic cell types of the endocrine pancreas, including the ß-cells, remains unclear. This, together with uncertainty about whether some key regulators act in progenitors or postmitotic ß-cells, or in both, obscures the sequence of regulatory events converting pancreatic progenitors into fully differentiated, insulin-expressing ß-cells.

Insulin-like peptides (Ilps) have been identified in many vertebrate and invertebrate species, suggesting that they evolved prior to a specialised endodermally derived pancreas [7,8]. In *Drosophila*, seven differentially expressed Ilps have been identified as orthologues of mammalian insulins and/or insulin-like growth factors [9]. Four of the Ilps (Ilp1, Ilp2, Ilp3, Ilp5) are coexpressed in a small population of median neurosecretory cells (mNSCs) in the brain that share a common progenitor lineage [10]. This provides a source of Ilps that can be secreted into the circulating haemolymph to promote insulin signalling in many different target tissues. Several elegant studies have harnessed the power of *Drosophila* genetics to demonstrate that insulin signalling plays important functions in regulating organismal growth, cell differentiation, glucose homeostasis, lipid metabolism, lifespan, and fertility [11–16]. *Drosophila* also promises to provide a powerful system for investigating how insulin production is regulated. Progress in this area, however, remains limited by the lack of information on the key regulators that generate insulin-producing cells from their progenitors.

During central nervous system (CNS) development, early differentiating neurons extend pioneer tracts that prefigure the major axonal tracts and guide subsequent nerve growth [17–19]. The presence of these pioneer (or primary) neuron populations is a conserved feature of CNS development and, remarkably, the early axonal scaffolds of the insect ventral nerve cord (VNC) and the vertebrate spinal cord are very similar [17,20–22]. The importance of pioneer tracts is well established: targeted ablation or blocked differentiation of pioneer neurons results in defective fasciculation and misrouting of follower axons [23–25]. As pioneer neurons have been observed to undergo apoptosis in the embryo, it has been proposed that once they have established the axonal scaffold, they have no further function [24,26,27]. In the embryonic *Drosophila* VNC, however, identified dMP2 pioneer neurons only die in anterior segments, leaving a surviving posterior subpopulation. Cell death in posterior dMP2 neurons is normally prevented by the Hox protein Abdominal-B (Abd-B), which represses the pro-apoptotic genes *reaper* (*rpr*) and *grim* [28].

The persistence of some dMP2 neurons raises the possibility that, either there is a longer-lasting requirement for axonal scaffolding functions in posterior than in anterior segments, or that surviving dMP2 neurons perform additional, as yet uncharacterised, roles. Here, we show that surviving dMP2 neurons express the insulin-like peptide Ilp7 at late stages, thus identifying a posterior neural source of insulin distinct from the brain mNSCs. We use axonal tracing and genetic cell ablations to show that dMP2 neurons mature and differentiate into insulin-producing visceral neurons after they are no longer required for axonal scaffolding. Then, making use of the well-defined dMP2 cell lineage and postmitotic mutant rescue experiments, we identify five regulators of the late neuronal programmes for cell survival and Ilp7 expression. This study reveals, for the first time to our knowledge, similarities between the insulinergic genetic programmes of the *Drosophila* CNS and the mammalian pancreas.

## Results

### Posterior dMP2 Neurons Express Ilp7

**Figure 1:**
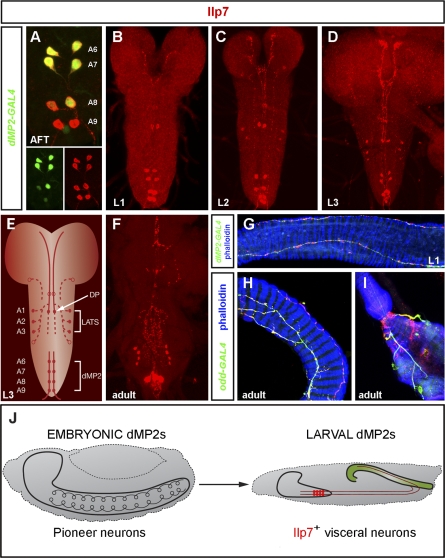
dMP2 Pioneer Neurons Become Insulinergic Visceral Neurons That Innervate the Larval and Adult Hindgut Muscles (A) Co-expression of *dMP2-GAL4* and Ilp7 in the four posterior pairs of Ilp7-expressing neurons in an air-filled tracheae (AFT) embryo. *dMP2-GAL4* expression in A8/A9 is weak or absent. (B–D) Developmental expression of Ilp7. (B) Ilp7 expression in a single dorsal pair and the four ventral, posterior pairs of dMP2 neurons in a first-instar larva. (C) In a second-instar larval CNS, Ilp7 is also expressed in one lateral neuron per hemisegment in two or three abdominal segments. (D) Robust Ilp7 expression in these three cell types in a third-instar larval CNS. (E) Cartoon summarizing the mature Ilp7 expression pattern in a third-instar larva. Besides the eight posterior dMP2 neurons in A6-A9, a dorsal pair (DP) in A1 and the three lateral pairs in A1–A3 typically express Ilp7 (LATs). Weak Ilp7 immunoreactivity is occasionally observed in a fourth lateral neuron per hemisegment in A4, a more anterior dorsal pair, and a neuron located at base of each brain hemisphere (empty circles). dMP2 axons exit in the posterior nerve, whereas DP processes extend anteriorly towards the *pars intercerebralis* and terminate near the insulin-producing mNSCs (Figure S2). LAT processes are not always apparent, but they seem to project towards the midline. (F) The Ilp7 larval expression pattern is maintained in the adult abdominal ganglion, although some remodelling appears to occur. (G) dMP2 axons (green) on the larval hindgut (muscles in blue). The nerve endings that contact the hindgut have several varicosities, where Ilp7 (red) release may occur. More complex Ilp7-positive dMP2 fibres innervate the adult hindgut (anterior intestine [H] and rectum [I]), as revealed by the presence of *odd^GAL4^*-positive axons (green) in close contact with the adult hindgut muscle (blue). (J) From pioneer neuron to insulin-producing visceral neuron. At the end of embryogenesis, anterior dMP2 neurons undergo apoptosis. The expression of the Hox gene Abd-B in posterior segments prevents this death, and posterior dMP2 neurons exit the VNC and become insulin-producing visceral neurons. Genotypes: (A) *dMP2-GAL4/UAS-nmEGFP*; (B–D and F) *y w*; (G) UAS*-CD8-GFP/+; dMP2-GAL4/+*; and (H and I) *odd^GAL4^,UAS-GFP/UAS-CD8-GFP*.

To investigate potential late roles for the surviving posterior dMP2 pioneer neurons, we searched the literature for candidate factors with restricted expression in a single neuron in posterior hemisegments. A previous study noted that Ilp7 has a similar segmentally restricted pattern of mRNA expression in the VNC [9]. After confirming the predicted Ilp7 mRNA sequence by reverse transcriptase-PCR (see Text S1 for details), we generated antibodies recognizing either the A or the B chain of the Ilp7 peptide (Figure S1D). These both show that the spatial expression pattern of Ilp7 peptide is similar to that of Ilp7 RNA (Figure S1A–S1C). Henceforth, the antibody against the Ilp7 B chain is used in this study. Ilp7 peptide is first detected just prior to larval hatching, in late stage 17 embryos with air-filled tracheae (AFT). A dMP2-specific *GAL4* driver [28] was used to reveal that Ilp7 is strongly activated at this stage in the single dMP2 neuron in each of the A6-A9 hemisegments (Figure 1A). Thus, in addition to weakly expressing the widely distributed neuropeptide proctolin [28], dMP2 neurons specifically and strongly express Ilp7 peptide. We hereafter refer to these Ilp7-producing neurons as insulinergic. Once activated, Ilp7 is strongly and stably expressed in postmitotic dMP2 neurons throughout the three larval instars (L1–L3) and also into adulthood (Figure 1B–1F). Ilp7 is also weakly and dynamically expressed in a few other neurons located anterior to A6 (Figures 1B–1F). In contrast to four other Ilps, it is not expressed in the brain mNSCs. However, Ilp7 neurites, likely originating from a single dorsal pair of Ilp7-expressing neurons (DP, Figure 1E), project close to the insulin-expressing mNSCs (Figure S2).

The dMP2 neuron is produced from the MP2 precursor, an atypical neuroblast that divides once and only once to generate a two-cell lineage in which the differential activation of Notch signalling determines sibling identity [29–32]. Combining this previous lineage information with our Ilp7 analysis indicates that the MP2 precursor division in A6–A9 generates an Ilp7^+^ neuron (dMP2), in which Numb suppresses Notch signalling, and an Ilp7^−^ Numb^−^ sibling neuron (vMP2) with active Notch signalling.

### Ilp7-Expressing dMP2 Neurons Innervate the Larval and Adult Hindgut

In addition to the neural Ilp7 expression, we observed strong Ilp7 immunoreactivity in the larval and adult hindgut. Costaining with dMP2 drivers indicates that the axons of posterior dMP2 neurons exit the VNC in the posterior nerve at embryonic stage 16 and innervate the hindgut, forming two fascicles that extend on opposite sides of the hindgut in an aboral to oral direction during larval stages (Figure 1E and 1G). Ilp7-positive nerve swellings are apparent throughout the length of these dMP2 fibres, consistent with *en passant* release sites over the circular muscles of the hindgut. Pre-incubation of Ilp7 antibodies with dissected hindguts prior to fixation reveals extracellular expression of mature Ilp7 peptide at dMP2 terminals (Figure S1E–S1F'). By the adult stage, hindgut Ilp7-positive fibres have become arborized with branches innervating both the anterior intestine and the rectum (Figure 1H and 1I). Besides the CNS and hindgut, we did not detect Ilp7 immunoreactivity in any other larval tissues.

Our analysis thus far reveals that, following a segment-specific burst of cell death, surviving posterior dMP2 neurons undergo a transition to become insulin-producing visceral neurons that innervate the larval and adult hindgut (Figure 1J).

### Pioneer Function and Insulinergic Identity Are Temporally Separated

**Figure 2:**
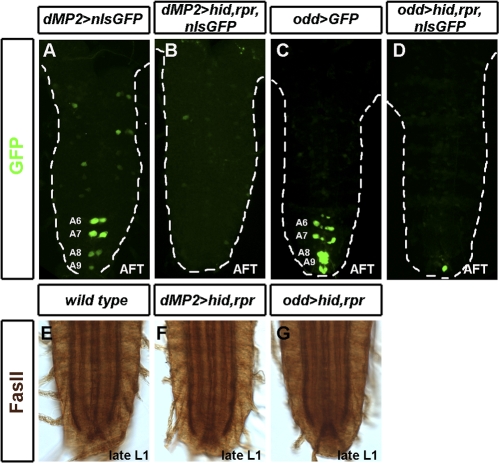
Pioneer Neurons Are Not Required to Maintain the Larval Axonal Tracts (A) Wild-type expression of *dMP2-GAL4* in dMP2 neurons. Expression in A8/A9 is weak or absent. (B) No dMP2 neurons remain at the end of embryogenesis following expression of the cell death activators *rpr* and *hid* from *dMP2-GAL4*. (C) Wild-type expression of *odd^GAL4^* in dMP2 and MP1 pioneer neurons in air-filled tracheae embryos at the end of embryogenesis. (D) Complete absence of dMP2 and MP1 pioneer neurons at the end of embryogenesis following expression of *rpr* and *hid* from *odd^GAL4^*. (E–G) Fasciclin II staining of larval longitudinal connectives is unaffected by the ablation of dMP2 neurons alone (F) or dMP2 and MP1 neurons (G) in late embryos. Genotypes: (A) *dMP2-GAL4/UAS-nmEGFP*; (B and F) *UAS-hid,UAS-rpr/Y; dMP2-GAL4/UAS-nmEGFP*; (C) *odd^GAL4^,UAS-GFP/+*; (D and G) *UAS-hid,UAS-rpr/Y; odd^GAL4^,UAS-GFP/+*; and (E) *w^1118^*.

We first detect Ilp7 expression in surviving posterior dMP2 neurons more than 5 h after their anterior counterparts have undergone apoptosis [23,28]. To determine whether Ilp7-expressing dMP2 neurons are retained because they are required for some late pioneer-like role in posterior fascicle maintenance, we used *dMP2-GAL4* to express the cell death activators *hid* and *rpr* (Figure 2A and 2B). Ablation of posterior dMP2 neurons at stage 17 did not disrupt the integrity of the major Fasciclin II-positive larval longitudinal connectives (Figure 2E and 2F). Nevertheless, as different pioneer neurons can function synergistically in longitudinal tract formation [23], we next ablated two types of pioneer neuron simultaneously. Using *odd-GAL4* [28,33] to express *hid* and *rpr,* we were able to co-ablate dMP2 and MP1 pioneer neurons during embryonic stages 15–17 (Figure 2C and 2D). Despite loss of all dMP2 and MP1 pioneer neurons from stage 17 onwards, the Fasciclin II-positive longitudinal tracts remained intact at late L1 stages (Figure 2G). Together, the ablation experiments reveal that after posterior dMP2 neurons have fasciculated with later-differentiating neurons to form mature axon bundles and have activated Ilp7 expression, they are no longer required for fascicle maintenance. Therefore, pioneer function and insulinergic activity correspond to distinct early and late steps during the postmitotic maturation of the dMP2 neuron.

### Retrograde BMP Signalling Promotes Ilp7 Expression

**Figure 3:**
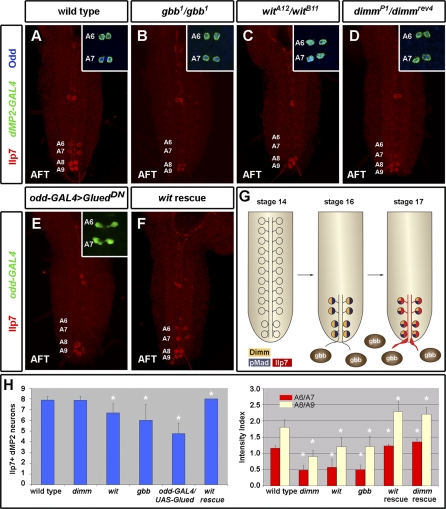
Retrograde Gbb/Wit Signalling and Dimm Regulate Ilp7 Expression (A) Wild-type embryos at the air-filled tracheae (AFT) stage express Ilp7 (red), Odd (blue), and *dMP2-GAL4* (green) in A6 to A9 dMP2 neurons. In *gbb* mutants (B) or *wit* mutants (C), Ilp7 expression in dMP2 neurons is absent or reduced. (D) In *dimm* mutant embryos, Ilp7 expression levels are reduced. (E) Blocking retrograde axonal transport cell-autonomously in dMP2 neurons results in absent or reduced Ilp7 expression. dMP2 neurons are otherwise correctly specified in all these mutants, as revealed by their expression of *dMP2-GAL4* and Odd (B–E, insets). (F) Postmitotic, cell-autonomous expression of *wit* in dMP2 neurons restores Ilp7 expression levels. (G) Acquisition of neuropeptidergic fate precedes Ilp7 expression. After their pioneer function, anterior dMP2 neurons die, whereas posterior dMP2 neurons begin to express *dimm*, exit the VNC, receive a *gbb* signal that leads to Mad phosphorylation, thus allowing them to express Ilp7. (H) Quantification of phenotypes. The number of posterior dMP2 neurons expressing Ilp7 in *wit*, *gbb*, and *odd^GAL4^/UAS-Glued* mutants at the end of embryogenesis is significantly reduced when compared to the wild-type (t-test, *p* < 0.001, *n* > 9 for all). Ilp7 is more frequently absent from A6/A7 than from A8/A9 segments in *wit*, *gbb*, and *dimm* mutants, probably because a general reduction in expression appears more pronounced in the weaker-expressing segments. dMP2 postmitotic expression of Wit in *wit* mutants significantly rescues this phenotype (*p* < 0.0001, *n* = 10). In first-instar larvae, Ilp7 intensity levels remain low (*p* < 0.01, *n* > 6 for all). dMP2 postmitotic expression of Wit in *wit* mutants or Dimm in *dimm* mutants significantly rescues this phenotype (*p* < 0.0001, *n* > 6 for all). The intensity index for A6/A7 and A8/A9 was quantified separately because the latter segments always express higher levels of Ilp7. Genotypes: (A) *UAS-CD8-GFP/+; dMP2-GAL4/+*; (B) *gbb^1^/gbb^1^*; *UAS-CD8-GFP/dMP2-GAL4*; (C) *UAS-CD8-GFP/+; dMP2-GAL4,wit^A12^/wit^B11^*; (D) *dimm^P1^/dimm^rev4^; UAS-CD8-GFP/dMP2-GAL4*; (E) *odd^GAL4^,UAS-GFP/UAS-Glued^DN^*; (F) *UAS-wit,wit^B11^/dMP2-GAL4, wit^A12^*; and (H) as above, plus *dimm* rescue is *dimm^rev4^/dimm^P1^; UAS-dimm/dMP2-GAL4*.

We then explored whether cell extrinsic factors might regulate the dMP2 insulinergic programme. Expression of the neuropeptide FMRFamide (FMRFa) requires a retrograde signal from Glass-bottom boat (Gbb), a bone morphogenetic protein (BMP) family growth factor [34,35]. This activates the Wishful-thinking (Wit) receptor in neurons, leading to the phosphorylation and consequent activation of Mothers against dpp (Mad). Although pMad activation is an absolute requirement for expressing FMRFa, no other *Drosophila* neuropeptides or neurotransmitters have yet been shown to be regulated in this manner. As Mad is also activated in posterior dMP2 neurons around the time when Ilp7 is first expressed (Figure 3G) [28], we examined Ilp7 expression in BMP pathway mutants. Reduced or absent Ilp7 expression was observed in *gbb* mutants (Figure 3B), *Mad* mutants (Figure S3E and S3F), *saxophone* (*sax*) mutants (Figure S4), and upon cell-autonomous interference with retrograde axonal transport using the dominant-negative dynactin, P150/Glued (*UAS-Glued^DN^*, Figure 3E). In *wit* mutants, we observed that Ilp7 expression is consistently reduced or absent in late embryos and early L1 larvae (Figure 3A, 3C, and 3H, *p* < 0.001). Even by the end of L1, Ilp7 intensity levels remain significantly lower than those of stage-matched wild-type larvae (Figure 3H, *p* < 0.01). Ilp7 expression is likely to be regulated at the transcriptional level by *wit*, *gbb*, and *Mad* as loss-of-function mutants show altered expression of Ilp7 RNA (Figure S3A–S3F). Cell-autonomous reintroduction of *wit* in dMP2 neurons rescues Mad activation and Ilp7 expression, thus demonstrating that the insulinergic programme requires BMP signal transduction in a cell-autonomous and postmitotic manner (Figure 3F and 3H, *p* < 0.001). In contrast to Ilp7 expression, dMP2 early specification and segment-specific survival are not affected in P150/Glued or BMP pathway mutants (Figures 3A–3E,insets, S5, and S6). Hence, retrograde BMP signalling does not provide a general dMP2 specification or survival signal but is required, at late postmitotic stages, to promote Ilp7 expression.

### Postmitotic Dimmed Activity Promotes Ilp7 Expression

We next investigated the identity of the cell-intrinsic transcription factors specifying the late postmitotic programme for insulinergic activity. The basic helix-loop-helix factor Dimmed (Dimm), the *Drosophila* orthologue of vertebrate Mist1, is an important regulator of neuroendocrine cell differentiation [36]. Dimm is first expressed in dMP2 neurons when apoptosis is underway in anterior segments, several hours before the onset of detectable Ilp7 expression (Figure 3G) [37]. Although many aspects of dMP2 differentiation are normal in *dimm* mutants, including segment-specific death (Figures 3D, inset, and S5C), Ilp7 peptide expression is significantly downregulated (Figure 3A, 3D, and 3H, *p* < 0.01). Dimm regulation of Ilp7 is not confined to the level of peptide processing as Ilp7 RNA is also downregulated in *dimm* mutants (Figure S3G and S3H). Ilp7 levels remain abnormally low in *dimm* mutants throughout development, and this phenotype can be reversed (to higher Ilp7 levels than those of wild-type larvae) by postmitotic, cell-autonomous expression of *dimm* in dMP2 neurons using *dMP2-GAL4* (Figure 3H, *p* < 0.0001). Thus, Dimm expression not only precedes but is also required for high-level Ilp7 production.

### Hb9 Functions Sequentially to Promote dMP2 Apoptosis and Ilp7 Expression

**Figure 4:**
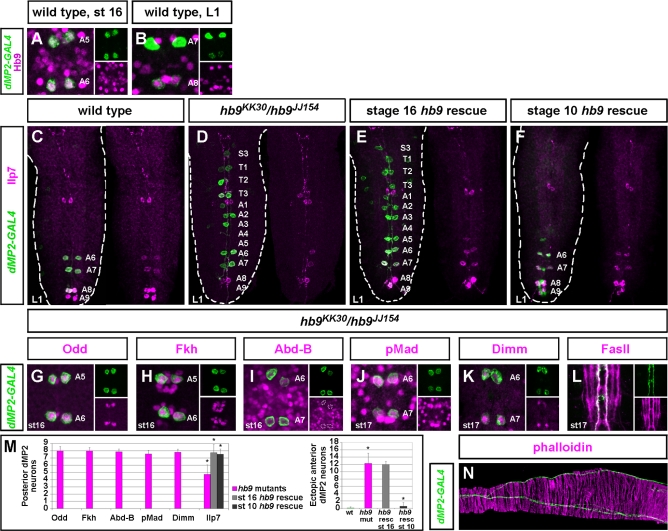
*hb9* Sequentially Regulates dMP2 Apoptosis and Ilp7 Expression (A) Hb9 expression in anterior and posterior dMP2 neurons before anterior apoptosis. (B) Hb9 persists in A8 and A9 larval dMP2 neurons, but it is downregulated from A6 and A7 dMP2s. (C) Wild-type, Ilp7-expressing dMP2 neurons in posterior segments in an early L1 CNS. (D) In *hb9* mutants, anterior dMP2 neurons fail to undergo apoptosis. Neither posterior dMP2 neurons nor ectopic anterior dMP2s express Ilp7. (E) Postmitotic, cell-autonomous expression of *hb9* from stage 16 in dMP2 neurons using *dMP2-GAL4* restores Ilp7 expression levels, but not apoptosis. A9 expression is not rescued, as *dMP2-GAL4* is rarely expressed in this segment. (F) Expression of *hb9* in dMP2 neurons from stage 10 rescues both Ilp7 expression and anterior apoptosis. (G–L and N) Other aspects of dMP2 specification are unaffected in *hb9* mutants. Anterior and posterior dMP2 neurons express early markers, such as Odd and Fkh (G and H, respectively). Unusually, Odd expression was weaker in anterior segments and was often absent from the S3, T1, and T2 anterior-most segments at stage 16, even though *dMP2-GAL4* and Fkh were present (unpublished data). Posterior dMP2 neurons express Abd-B (I). In late embryos, posterior dMP2 neurons express pMad (J) and Dimm (K), and exit the VNC in the correct nerve (L). Abnormal, lateral projections were only rarely observed (5% of posterior segments, unpublished data). (N) dMP2 axons innervate the hindgut correctly. (M) Quantification of *hb9* phenotypes. In the rescue of Ilp7 with *dMP2-GAL4*, Ilp7 expression was only assessed in *GAL4+* dMP2s, as assessed by *UAS-CD8-GFP* expression, given that *dMP2-GAL4* is not always expressed in A8, and it is rarely expressed in A9. For clarity, the total percentage of Ilp7-expressing/*dMP2-GAL4*-expressing posterior neurons per VNC has been recalculated for a final number of eight neurons, in order to compare it to the wild-type Ilp7 expression. *n* > 10 VNCs for each marker. Asterisks denote significance (t-test *p* < 10^−5^ or less) when *hb9* mutants were compared to wild-type CNSs, or when rescued CNSs were compared to *hb9* mutants. Genotypes: (A–C) *UAS-CD8-GFP/+; dMP2-GAL4/+*; (D, G–L, and N) *UAS-CD8-GFP; hb9^JJ154^/dMP2-GAL4,hb9^KK30^*; (E) *UAS-CD8-GFP/+*; *UAS-hb9*,*hb9* *^JJ154^*/*dMP2-GAL4*,*hb9* *^KK30^*; and (F) *odd^GAL4^*,*UAS-GFP*; *UAS-hb9*,*hb9* *^JJ154^* */hb9* ^*KK30*^.

In *Drosophila*, the homeodomain transcription factor Hb9 is expressed in a subset of postmitotic motor neurons, where it regulates axonal pathfinding [38,39]. Interestingly, embryos lacking the cell death activators *hid*, *grim*, and *rpr* contain increased numbers of Hb9-positive neurons, suggesting that Hb9 might also promote neuronal cell death [40]. Both anterior and posterior dMP2 neurons express Hb9 from embryonic stage 10–11, soon after they become postmitotic [38]. Hb9 expression in dMP2 neurons is maintained throughout embryogenesis but, by first-instar stage, it is only apparent in dMP2 neurons of A8 and A9 (Figure 4A and 4B). Examination of dMP2 specification in *hb9* mutants revealed two distinct phenotypes: 68.3% of anterior dMP2 neurons fail to undergo apoptosis (Figure 4C, 4D, and 4M), and 40.8% of posterior dMP2 neurons (as well as 100% of ectopic anterior counterparts) fail to express Ilp7 (Figures 4C, 4D, 4M, and S3J). Reintroduction of *hb9* from embryonic stage 16 onwards using *dMP2-GAL4* rescued Ilp7 expression (96.7%, Figure 4E and 4M) but failed to rescue anterior apoptosis (66.7% of anterior dMP2 neurons persist in early first-instar larvae, Figure 4E and 4M). This experiment uncouples the dual roles of Hb9 in cell death and Ilp7 expression and indicates that late cell-autonomous activity of Hb9 is sufficient to activate Ilp7. We then reintroduced Hb9 in dMP2 neurons using *odd-GAL4*, which is expressed in dMP2 neurons from embryonic stage 10 onwards, much earlier than *dMP2-GAL4*. This efficiently rescued both anterior apoptosis (96.9% of anterior dMP2 neurons underwent apoptosis by early first-instar) and Ilp7 expression (94.1% of posterior dMP2 neurons were Ilp7-positive, Figure 4F and 4M). We thus conclude that the pro-apoptotic activity of *hb9* is required at an earlier stage of postmitotic dMP2 maturation than its function in Ilp7 activation.

To establish whether properties of dMP2 other than apoptosis and Ilp7 expression are also regulated by Hb9, we examined a number of markers that define different stages of dMP2 specification. Early dMP2 markers such as Odd-skipped (Odd) and Fork Head (Fkh, see next section) remain expressed in *hb9* mutants in both anterior and posterior dMP2 neurons (Figure 4G, 4H, and 4M). At stage 16, the posterior dMP2-specific marker Abd-B is also retained (Figure 4I and 4M). By stage 17, dMP2 neurons in *hb9* mutants are still able to express Dimm and activated Mad, project axons posteriorly along the most medial fascicle, and exit the VNC to innervate the hindgut correctly (Figure 4J–4N). Thus, Hb9 is not a general postmitotic regulator of all aspects of dMP2 differentiation. Instead, it promotes apoptosis and Ilp7 expression in a cell-autonomous and sequential manner (Figure S8).

### Fkh Regulates Apoptosis, Ilp7 Expression, and Other Aspects of dMP2 Late Differentiation

**Figure 5:**
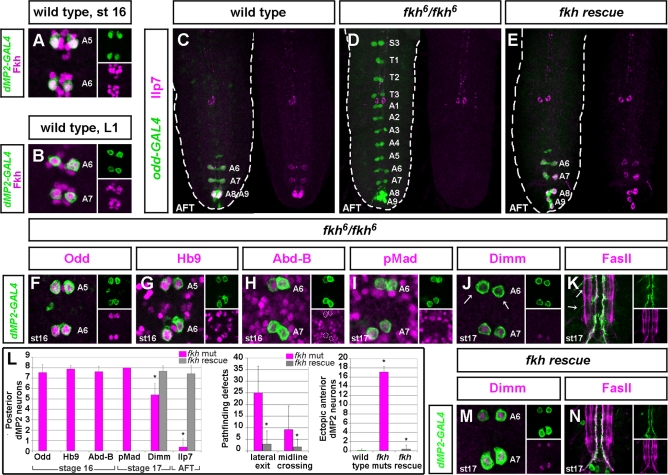
*fkh* Regulates All Aspects of Late dMP2 Identity (A) Fkh expression in both anterior and posterior dMP2 neurons precedes apoptosis and Ilp7 expression. (B) Fkh expression persists in larval dMP2 neurons. (C) Wild-type, Ilp7-expressing dMP2 neurons in posterior segments in late embryo. (D) In *fkh* mutants, almost all anterior dMP2 neurons fail to undergo apoptosis. Neither posterior dMP2 neurons nor these ectopic anterior dMP2s express Ilp7. (E) Cell-autonomous expression of *fkh* in dMP2 neurons restores anterior apoptosis and posterior Ilp7 expression. (F–K) In *fkh* mutants, the transition from dMP2 pioneer to Ilp7 neuropeptidergic neuron is stalled. dMP2 neurons express early markers in every segment, such as Odd and Hb9 (F and G). Posterior dMP2 neurons also express Abd-B (H). Later, activation of Mad occurs, but is often delayed (I) and Dimm expression is delayed or absent (arrows). (K) Some late pathfinding defects are apparent, such as premature exit of dMP2 axons laterally. Gut innervation could not be assessed because of the absence of hindgut in *fkh* mutants [71]. (L) Quantification of observed phenotypes in posterior dMP2 neurons. Left graph: expression of dMP2 markers in posterior dMP2 neurons in mutant and rescued CNSs. Middle graph: Number of segments with pathfinding defects per mutant VNC. Right graph: ectopic anterior survival in mutant and rescued CNSs. Asterisks indicate that mutant numbers are significantly different from wild-type, or that rescue numbers are significantly different from mutant ones (*p* < 0.001 or less for all t-tests except for midline crossing, where *p* = 0.05, *n* > 10 for each genotype). (M and N). Rescue of Dimm expression and pathfinding defects upon cell-autonomous expression of *fkh* in dMP2 neurons in *fkh* mutants. Genotypes: (A and B) *UAS-CD8-GFP/+; dMP2-GAL4/+*; (C) *odd^GAL4^,UAS-GFP/+*; (D) *odd^GAL4^,UAS-GFP/+; fkh^6^/fkh^6^* ; (E) *odd^GAL4^,UAS-GFP/+; UAS-fkh,fkh^6^/fkh^6^*; (F–K) *UAS-CD8-GFP; CY27-GAL4,fkh^6^/fkh^6^*; and (M and N) *UAS-CD8-GFP; CY27-GAL4,fkh^6^/UAS-fkh,fkh^6^.* The routinely used *dMP2-GAL4* driver is not expressed in *fkh* mutants, hence the choice of *CY27-GAL4* to identify dMP2 neurons up to stage 17 or *odd^GAL4^* in late embryos with air-filled tracheae where *CY27-GAL4* expression is no longer restricted to dMP2 neurons.

We next examined the expression pattern of the winged-helix/forkhead box transcription factor Fkh, which has not been well characterised in the *Drosophila* CNS. We find that Fkh is expressed in segmentally repeated clusters of midline neurons, including dMP2, vMP2, MP1 neurons, and the VUM interneurons (Figure 5A and unpublished data). Within the MP2 lineage, Fkh is first expressed in the MP2 neuroblast at stage 9–10 and continues to be expressed in both the dMP2 and vMP2 daughters throughout embryonic and larval stages (Figure 5B). In *fkh* mutants, 95% of anterior dMP2 neurons fail to undergo apoptosis, and 95.3% of posterior dMP2 neurons (and 100% of ectopic anterior counterparts) fail to express Ilp7 (Figures 5C, 5D, 5L, and S3I). Both of these dramatic phenotypes could be rescued to near wild-type levels by reintroducing Fkh under *odd-GAL4* regulation, indicating a cell-autonomous requirement for promoting dMP2 apoptosis and Ilp7 expression (Figure 5E and 5L).

The Fkh requirement does not extend to all features of dMP2 specification as *fkh* mutant dMP2 neurons still retain expression of some early dMP2 markers such as Odd, Hb9, and Abd-B at stage 15–16 (Figure 5F–5H and 5L). However, later events such as Mad activation and Dimm expression are delayed or blocked (Figure 5I, 5J, and 5L). Also, at stage 17, dMP2 axons often exit the VNC prematurely or, occasionally, cross the midline (Figure 5K and 5L). These phenotypes could all be rescued by reintroducing Fkh in dMP2 neurons in a cell-autonomous manner (Figure 5L–5N).

Thus, dMP2 neurons are initially specified with the correct early postmitotic identity in an Hb9- and Fkh-independent manner. Their late differentiation, however, requires inputs from both transcription factors. Removing either factor blocks segment-specific survival and subsequent Ilp7 expression. However, whereas Hb9 specifically affects Ilp7 expression, Fkh acts as a postmitotic progression factor required for most aspects of late dMP2 identity (Figure S8).

### The Hox Protein Abd-B Is Specifically Required for Ilp7 Expression

**Figure 6:**
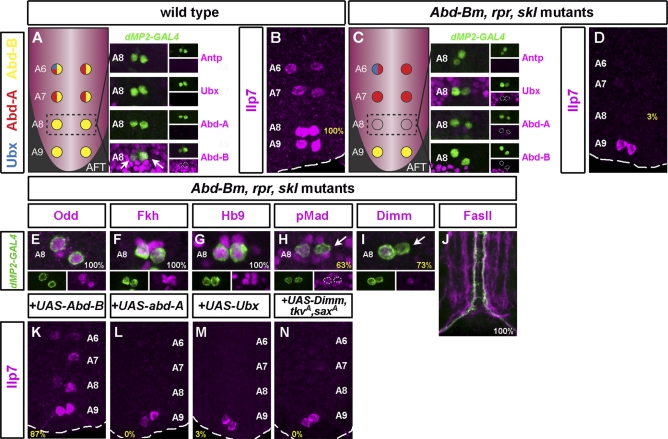
*Abd-B*, but Not Other Hox Genes, Is Required for Ilp7 Expression (A) Hox code in wild-type posterior dMP2 neurons at the onset of Ilp7 expression. While A8 and A9 only express Abd-B, A6 and A7 co-express Abd-A and Abd-B. Ubx expression is occasionally detected in A6. Note no Hox expression in A8 other than Abd-B. (B) Wild-type Ilp7 expression in A6–A9. (C) Hox code of posterior dMP2 neurons in *Abd-Bm, rpr, skl* mutants: A6 and A7 express Hox genes other than Abd-B (namely Abd-A and, occasionally, Ubx), A8 is a Hox-free dMP2, and A9 expresses Abd-B, like in the wild-type. (D) Ilp7 immunoreactivity in *Abd-Bm, rpr, skl* mutants is only apparent in A9: the only segment with Abd-B expression. (E–J) Other aspects of dMP2 identity are generally unaffected by the absence of Hox expression. A8 dMP2 neurons express early markers, such as Odd (E), Fkh (F), and Hb9 (G). Expression of pMad and Dimm is occasionally affected (H and I, arrows). No pathfinding defects are apparent (J). (K) Postmitotic, cell-autonomous expression of *Abd-B* in dMP2 neurons rescues Ilp7 expression in *Abd-B, rpr, skl* mutants. By contrast, expression of other Hox genes does not rescue (L and M). (N) No Ilp7 rescue by restoring wild-type Dimm and pMad expression in *Abd-B, rpr, skl* mutants*.* Genotypes: (A and B) *dMP2-GAL4/UAS-nmEGFP*; (C and D) *UAS-nmEGFP/+; dMP2-GAL4,H99,AbdB^M5^/XR38,AbdB^M5^*; (E–J) *UAS-CD8-GFP/+; dMP2-GAL4,H99,AbdB^M5^/XR38,AbdB^M5^*; (K) *UAS-Abd-B/+; dMP2-GAL4,H99,AbdB^M5^/XR38,AbdB^M5^*; (L) *dMP2-GAL4,H99,AbdB^M5^/UAS-abd-A,XR38,AbdB^M5^*; (M) *UAS-Ubx/+; dMP2-GAL4,H99,AbdB^M5^/XR38,AbdB^M5^*; and (N) *UAS-tkv^A^,UAS-sax^A^/+; dMP2-GAL4,H99,AbdB^M5^/UAS-Dimm,XR38,AbdB^M5^*.

Members of the conserved Hox gene family regulate segment-specific aspects of cell fate (reviewed in [41,42]). The Hox protein Abd-B is known to promote posterior dMP2 survival [28]. We next addressed whether Abd-B or other Hox proteins might also regulate aspects of dMP2 differentiation. At early stage 17, prior to the onset of Ilp7 expression, posterior dMP2 neurons in A6–A7 coexpress the Hox proteins Abdominal-A (Abd-A) and Abd-B (and, occasionally in A6, Ultrabithorax [Ubx]), whereas in A8–A9 they express Abd-B only (Figure 6A). To test the involvement of Hox proteins in dMP2 differentation, we generated triple mutants simultaneously lacking the activity of Abd-Bm (the Abd-B isoform expressed in A6–A8 dMP2s) and the pro-apoptotic genes *rpr* and *sickle* (*skl*). In these triple mutants, posterior dMP2 neurons survive even though Abd-Bm activity is absent from A6–A8. In A6 and A7, dMP2 neurons still express Abd-A, A8 is Hox-free, and A9 retains expression of the Abd-Br isoform (Figure 6C) [28]. We examined Ilp7 peptide in these mutants and found it to be expressed in A9 but absent from A6–A8 (Figure 6D). Ilp7 RNA was also absent from A8 and reduced or absent from A6/A7 (Figure S3K). This indicates that Abd-B activity is required for Ilp7 expression and suggests that the other Hox proteins expressed in dMP2 neurons do not possess this function. To test this more directly, we reintroduced several Hox proteins in *Abd-B, rpr, skl* mutant dMP2 neurons postmitotically and cell-autonomously using *dMP2-GAL4*. In this context, Abd-B expression is able to rescue Ilp7 expression (87%, Figure 6K) but neither Abd-A (Figure 6L) nor Ubx (Figure 6M) are able to substitute for this function. Thus, Abd-B is required cell-autonomously for Ilp7 expression in posterior dMP2 neurons.

We then assessed whether Abd-B has a positive input into Ilp7 activation or whether it functions negatively in dMP2 neurons to repress more anterior Hox proteins [43]. We therefore examined the development of dMP2 neurons in A8: the Hox “ground-state” segment in *Abd-B*, *rpr*, *skl* mutants that lacks expression of any Hox genes. dMP2 generation and early specification in A8 occur normally, as revealed by their expression of Odd, Fkh, and Hb9 (Figure 6E–6G, respectively). Nevertheless, acquisition of late identity at stage 17 was partially disrupted as 27% of A8 dMP2 neurons fail to express Dimm (Figure 6I) and 37% of them fail to activate BMP signalling (Figure 6H). These low-expressivity effects appear unlikely to account for the 100% absence of Ilp7 as restoration of Dimm and BMP signalling was unable to rescue Ilp7 expression in triple-mutant dMP2 neurons in A8 (Figure 6N). Although we could not assess pathfinding in an A8 segment-specific manner, no ectopic dMP2 projections were observed (Figure 6J).

In summary, while early dMP2 specification is largely Hox-independent, Ilp7 expression is under postmitotic Hox control at two levels: Abd-B promotes posterior dMP2 survival and subsequently plays a specific role in promoting Ilp7 expression (Figure S8).

### Misexpression of the Insulinergic Code Ectopically Activates Ilp7 in Some Neurons

**Figure 7:**
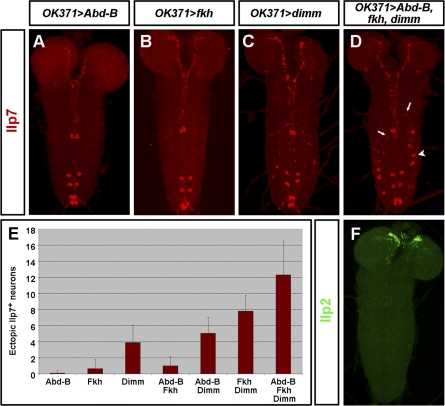
Coexpression of *dimm*, *fkh* and *Abd-B* Triggers Ectopic Ilp7 Activation in a Subset of Motor Neurons (A–C) Single misexpression of Abd-B (A), Fkh (B) or Dimm (C) in motor neurons has no or very limited effect. (D) Misexpression of Dimm, Fkh and Abd-B in motor neurons leads to strong ectopic Ilp7 activation in one motor neuron per hemisegment in A1–A7 (arrowhead) in a first-instar VNC. Weak Ilp7 activation is occasionally observed in several other motor neurons (arrows). (E) Quantification of Ilp7 activation. The identified Ilp7 regulators act in a combinatorial manner: the triple comisexpression triggers the highest number of Ilp7 ectopic neurons. This number is significantly higher than that of any double co-expression (*p* < 10^−8^ when compared to any of them). Co-expression of Dimm, Fkh significantly triggers more ectopic Ilp7 than Dimm (*p* < 10^−6^). The ectopic activation obtained with Dimm, Abd-B is not significantly different from that of Dimm alone (*p* = 0.2). *n* > 20 for all genotypes. (F) Misexpression of Dimm, Fkh and Abd-B in motor neurons does not trigger ectopic Ilp2 activation. Only endogenous Ilp2 is apparent in the brain mNSCs. Genotypes: (A) *OK371-GAL4/UAS-Abd-B*; (B) *OK371-GAL4/+; UAS-fkh/+*; (C) *OK371-GAL4/+; UAS-dimm/+*; and (D and F) *OK371-GAL4/UAS-Abd-B; UAS-dimm,UAS-fkh*.

To test the sufficiency of the transcription factors identified in this study, we attempted to reconstitute an ectopic “Ilp7 code” in other postmitotic neurons. To bypass embryonic lethality, we confined our misexpression to a subset of postmitotic neurons. *OK371-GAL4* was used to drive expression in about 30 postmitotic motor neurons per hemisegment [44]. We reasoned that, as motor neurons possess active BMP signalling and often express Hb9 [35,38,39], misexpression of Dimm, Abd-B, and Fkh in this neuronal type might be sufficient to reconstitute the Ilp7 code described here. When either Abd-B or Fkh were misexpressed in this motor neuron domain, no ectopic Ilp7 activation was observed (Figure 7A, 7B, and 7E). Misexpression of Dimm alone led to occasional, weak activation of Ilp7 in one cell per hemisegment (Figure 7C and 7E). In contrast, combinatorial misexpression of Dimm, Fkh, and Abd-B consistently triggered strong ectopic Ilp7 activation in one neuron per hemisegment in A1–A7 together with weaker ectopic expression in several other neurons (Figure 7D and 7E). This triple misexpression resulted in more Ilp7-positive ectopic cells than in any double misexpression (Figure 7E, *p* < 0.001), indicating that all three Ilp7 regulators contribute in a combinatorial manner to activate Ilp7. Interestingly, combinatorial misexpression of Dimm, Fkh, and Abd-B in motor neurons did not lead to any detectable ectopic expression of Ilp2, an insulin-like peptide known to be expressed in the brain mNSCs (Figure 7F), nor did we observe ectopic activation of other neuropeptides/peptide hormones such as FMRFa (Figure S7). Thus, the insulinergic code that we have identified appears to be specific for Ilp7 expression.

## Discussion

This study identifies an insulin-producing neuron and its cell lineage in *Drosophila*. It also uncovers four transcription factors and a retrograde signal that are required for this neuron to switch on expression of an insulin-like peptide. We now discuss the implications of these findings with respect to postmitotic neuronal maturation and the combinatorial transcription factor codes for neuronal death and insulin production.

### Postmitotic Maturation from Pioneer to Insulinergic Visceral Neuron

The observed death of some *Drosophila* pioneer neurons has been used to argue that their function is transient [24,27], but persistence in other cases suggested that, either they continue to play an axonal-scaffolding role, or that they adopt some other identity [27,45]. Our findings resolve this long-standing issue by clearly demonstrating that, for dMP2 neurons, the axonal scaffolding function is only transient. After this role is no longer required, surviving dMP2 neurons become insulinergic and innervate the hindgut. The other known innervation of the *Drosophila* gut occurs much more anteriorly, in the foregut and anterior midgut, from neuronal cell bodies located in the peripheral ganglia of the stomatogastric nervous system [46]. Unlike dMP2 neurons, however, the individual identities of the stomatogastric neurons and their cell lineages remain to be clearly defined. Thus, dMP2 neurons may provide a simple and well-characterised system for studies of the guidance cues involved in enteric innervation. Future studies, however, will be needed to determine the functions of Ilp7 in dMP2 neurons. It will be important to distinguish if this posterior neural source of insulin acts humorally to promote growth, like the more anterior brain mNSCs [15,16], or if it has more local effects in abdominal tissues. In this regard, the presence of Ilp7-expressing neurites in close proximity to the Ilp2-producing mNSCs is intriguing.

The transition from pioneer to neuroendocrine neuron is not unique to dMP2 neurons, as *Drosophila* MP1 pioneer neurons also become neuropeptidergic at larval stages [47]. In the grasshopper, segment-specific survival of pioneer neurons has also been reported [45], raising the possibility that they too may become neuroendocrine. Studies in other species, including vertebrates, will be needed to reveal the extent to which the linkage between pioneer and neuroendocrine functions is conserved. Identifying pioneer neurons with an “ancestral” neuroendocrine identity [48] in other phyla would lend further support to the proposal that pioneer neurons are highly conserved in evolution [17,20,49].

### Combinatorial Developmental Regulation of Neuronal Apoptosis

Apoptosis of postmitotic neurons is a widespread feature of normal VNC development [28,40], but few developmental regulators of core pro-apoptotic genes such as *grim*, *hid*, and *rpr* have been identified. This study uncovers roles for Fkh and Hb9. Hb9, at least, appears linked to cell death in neurons other than dMP2: in *Df(3L)H99* mutant embryos, where apoptosis is blocked, ectopic Hb9-positive RP motor neurons are observed in segments A7–A8 [40]. Hb9 is an important regulator of motor neuron identity in both *Drosophila* and vertebrates [50,51]. Our finding of a pro-apoptotic function for Hb9 in *Drosophila,* together with the neurotrophic requirement for motor neuron survival in vertebrates, raises the possibility that the same genetic programmes specifying the identities of motor neurons also sensitise them for postmitotic editing via apoptosis.

Hb9 and Fkh expression in many neurons that do not die suggests a combinatorial mechanism for the control of developmental apoptosis. One possibility is that several transcription factors function in combination to activate the core pro-apoptotic genes. Given the proposed role for Foxa proteins in chromatin accessibility [52], Fkh expression in dMP2 neurons may render the promoters of core pro-apoptotic genes responsive to activation by Hb9. An alternative but not mutually exclusive mechanism involves individual transcription factors activating different pro-apoptotic genes such that a combination of these would then be required to trigger neuronal death. For example, Hb9 could be required for *rpr/skl* but not *grim* expression. Some support for this idea comes from the observation that loss of *hb9* activity blocks *rpr/skl*-mediated death of dMP2 neurons but not the largely *grim*-dependent apoptosis of anterior MP1 neurons (unpublished data).

An important conclusion from this study is that the combinatorial transcription factor code controlling apoptosis partially overlaps with that regulating insulinergic identity (see next section). Thus, Fkh and Hb9 are both essential components of the codes for anterior apoptosis and also Ilp7 expression, illustrating that these transcription factors play surprising dual roles as pro-apoptotic and pro-differentiation factors within the same neuronal subtype. Importantly, our results also show that the segment-specific Hox protein Abd-B acts as a postmitotic switch, converting the pro-apoptotic Fkh^+^ Hb9^+^ code into an insulinergic Fkh^+^ Hb9^+^ Abd-B^+^ code.

### A Postmitotic Combinatorial Code for Insulinergic Identity

Three Ilp7 regulators (Hb9, Abd-B, and Fkh) are expressed at least 12 h before Ilp7 is first activated: from the time when the MP2 neuroblast exits the cell cycle. In the case of Hb9, we were able to uncouple two temporally separable functions. Early postmitotic expression of Hb9 is important for its death-activating function, whereas later expression suffices for activating Ilp7. Similarly, the Hox protein Abd-B generates a segment-specific neuropeptide pattern via postmitotic regulation of posterior dMP2 survival and also Ilp7 activation. As vertebrate neuropeptides are also expressed in restricted neuronal populations within specific rostrocaudal domains [53,54], they may be similarly regulated by Hox survival/neuroendocrine inputs. In the case of Fkh, it is required for many different aspects of the progression from the early to the late postmitotic dMP2 fate. Fkh expression is restricted to VNC midline neurons and its vertebrate orthologue Foxa2 functions in the differentiation of the floor plate and ventral dopaminergic and serotonergic neurons [55–57]. Thus, in both the *Drosophila* midline and its vertebrate counterpart, the floor plate [20,58], Fkh proteins play a conserved role in the differentiation of ventral neuronal subtypes.

**Figure 8:**
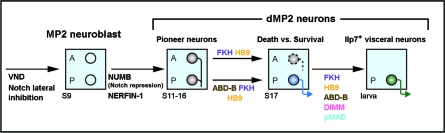
A Multi-Step Transcriptional Programme for Insulinergic Identity Cartoon summarizing the genes and transitions from the generation of the MP2 neuroblast to the late specification of Ilp7 visceral neurons. Lateral inhibition singles out the MP2 neuroblast, which also requires Vnd for its formation. Asymmetric localization of Numb to dMP2 neurons during MP2 mitosis represses Notch signalling and specifies dMP2 identity. Nerfin-1 is required for pioneer function. The later death/survival decision and expression of Ilp7 require the intrinsic and extrinsic factors identified in this study (gene names in colour). See text for details.

The other two dMP2 regulators identified in this study, Dimm and the BMP pathway, are switched on shortly before the onset of Ilp7 expression. The timing of onset of these two broad neuroendocrine regulators is likely to specify when Ilp7 is first activated, whereas the earlier factors Fkh, Hb9, and Abd-B may contribute more specifically to insulinergic identity. Together, the genetic and expression analyses in this study demonstrate that the combinatorial code of genetic inputs required for Ilp7 expression is assembled in a step-wise manner during postmitotic maturation (Figure 8). Importantly, this allows a subset of the components to be shared (such as Fkh and Hb9) between sequential neuronal programmes (survival and Ilp7 expression) without losing output specificity.

Two observations from this study indicate that insulinergic combinatorial codes can vary from cell-to-cell and also from one Ilp to another. First, none of the regulators of Ilp7 in dMP2 neurons appear to regulate it in DP neurons. Second, the dMP2 insulinergic code is sufficient to trigger ectopic expression of Ilp7 but not Ilp2 or other neuropeptides such as FMRFa. These findings suggest the existence of additional, as yet unidentified, insulinergic factors in DP neurons and also in the brain mNSCs where Ilp2 is expressed. Recent identification of the neural progenitor for these mNSCs [10] should facilitate characterization of the Ilp1/Ilp2/Ilp3/Ilp5 combinatorial codes and thus clarify the extent to which different insulinergic transcriptional programmes overlap.

### A Conserved Developmental Programme for Insulin Expression?

Our finding that an Ilp7-expressing neuron derives from the MP2 lineage reveals that at least some insulinergic regulators are similar in insects and mammals. Three apparent similarities may not be very insulin-specific but reflect more general processes shared by neural and endocrine programmes in many species. First, Notch signalling singles out the MP2 neuroblast and distinguishes its two progeny neurons, while in mammals, it limits pancreatic expression of the “proneural” gene *Ngn3* to prospective endocrine cells [6]. Second, the survival and pro-Ilp7 functions mediated by Abd-B in the dMP2 neuron could also have their postmitotic counterparts in ß-cells, either mediated by related Hox genes [59] or via another homeobox gene, *Pdx-1*, following its early input into pancreatic induction [6,60]. And third, Nerfin-1 is required for dMP2 pioneer function [61], while its mammalian orthologue Insm1/IA1 is important for pancreatic ß-cell specification [62].

Several more specific regulatory similarities exist between the insulinergic differentiation factors active in postmitotic dMP2 neurons (Figure 8) and pancreatic ß-cells [4,6]. For example, the role of *fkh* in dMP2 neurosecretory differentiation described in this study is similar to the functions of HNF3b/Foxa2 in islet maturation and insulin secretion [63,64]. In addition, mammalian Nkx2.2 is important for pancreatic ß-cell specification [65] and is known to activate transcription of the insulin regulator Nkx6.1: an important late event in ß-cell differentiation [6]. Intriguingly, the *Drosophila* orthologue of Nkx2.2, Vnd, is required for dMP2 formation [61,66]. Drosophila Nkx6.1, the orthologue of mammalian *Nkx6* (FlyBase name *HGTX*), is expressed by postmitotic dMP2 neurons [67], and it will be interesting to determine whether it too functions downstream of Vnd during Ilp7 regulation. Most strikingly, mammalian equivalents of two of the insulinergic inputs identified in this study, Hb9 and BMP signalling, are also required for several aspects of late ß-cell differentiation including the expression of Nkx6.1 and insulin [68–70]. Together, these insect-mammalian comparisons provide evidence that, although the cell types involved look very different, some of the genetic circuitry regulating insulin is conserved between arthropods and chordates. This suggests that the power of fly genetics can now be harnessed to identify additional mammalian regulators of neuroendocrine cell fates and insulin expression.

## Materials and Methods

### Fly stocks.

The following fly stocks were used: *dMP2-GAL4*, *UAS-nls-myc-EGFP* (referred to as *UAS-nmEGFP*), *Abd-B^M5^*, *UAS-Antennapedia*, *UAS-Ubx*, *UAS-abd-A*, UAS*-Abd-Bm* (referred to as *UAS-Abd-B*), *Df(3L)XR38* and *Df(3L)H99* deficiencies (referred to as *XR38* and *H99*, respectively) [28]; *wit^A12^*, *wit^B11^*, *UAS-tkv^A^*, *UAS-sax^A^*, *UAS-Glued^DN^*, *gbb^1^* [34]; *dimm^rev4^*, *dimm^P1^*, *UAS-dimm* [36]; *hb9^KK30^*, *hb9^JJ154^* [38]; *UAS-hb9* [39]; *fkh^6^* [71]; *Mad^10^*, *Mad^12^* [72]; *CY27-GAL4* [73]; *UAS-fkh* [74]; *odd^GAL4^, UAS-GFP* [33]; *UAS-CD8-GFP* [75]; *UAS-hid, UAS-rpr* [76]; *OK371-GAL4* [44]; and *Ilp2-GAL4* [16]. Mutants were maintained over *CyO,Act-GFP* or *TM3,Ser,Act-GFP* balancer chromosomes. *y w* or *w^1118^* or heterozygous mutant stocks were typically used as control. Crosses were maintained at 25 °C, except for *OK371* crosses, which were kept at 21 °C.

### Immunohistochemistry.

Immunolabelling was carried out as previously described [34]. Antibodies used were: rabbit α-Ilp7 (1:5,000, see below for details), rabbit α-Odd (1:1,000) [31], guinea pig α-Fkh (1:800) [77], rabbit α-Hb9 (1:2,000) [38], guinea pig α-Dimm (1:1,000) [78], rabbit α-pMad (1:2,000) [79], mouse α-Ubx mAb FP3.38 (1:20) [80], rat α-abd-A (1:400) [81], rat α-Ilp2 (gift of P. Leopold, 1:400), mouse and rabbit α-green fluorescent protein (GFP) (Molecular Probes, 1:1,000 and 1:2,000, respectively), rat α-mouse CD8a (Caltag, 1:100), mouse mAb 1D4 α-fascilin II (1:50), mouse α-Antennapedia mAb 4C3 (1:50), mouse α-Abd-B mAb 1A2E9 (1:20) (all from Developmental Studies Hybridoma Bank). Phalloidin-TX (Molecular Probes) was used at 1:1,000. FITC-, Rhodamine-Red-X-, and Cy5-conjugated secondary antibodies were obtained from Jackson Immunolabs and used at 1:200 (1:100 for the Cy5-conjugated antibody), and a Vectastain ABC kit was used for the detection of biotinylated anti-mouse antibody (Vector Laboratories, 1:1,000).

### Ilp7 antibody generation.

An antibody to Ilp7 was generated in rabbits against the synthetic peptide RERSQSDWENVWHQET. This sequence is within the predicted B chain and corresponds to amino acids 41–57 of the predicted Ilp7 protein. Peptide synthesis, antibody generation, and affinity purification were done by Eurogentec.

Details of a second antibody directed against the predicted A chain are available in Text S1.

### Confocal imaging and data acquisition.

Confocal images were obtained using a Leica SP5 confocal microscope. Where immunolabels were analysed for levels of expression, wild-type and mutant tissues were stained on the same slide. The intensity index used to quantify Ilp7 expression levels in *dimm*, *wit*, *gbb* mutants, and rescues (Figure 3H) was obtained as previously described [36]. Briefly, mutant and wild-type CNS were dissected, fixed, and processed together on polyLysine slides. They were imaged with exposure settings adjusted to optimize detection without saturating the signal, and identical settings were used for all preparations and genotypes. Mean pixel luminosity for the area covering the soma (S) was measured for each neuron using Adobe Photoshop. An adjacent area was sampled to measure the background signal (B). The intensity index was calculated as (S-B)/B. Statistical significance was calculated using two-tailed Student t-tests, assuming equal sample variance. Where appropriate, images were false-coloured for clarity, and red was converted to magenta for the benefit of colour-blind readers.

## Supporting Information

Figure S1 Ilp7 mRNA and Peptide Expression In situ hybridisation of Ilp7 mRNA in first- and third-instar larvae (A and B, respectively) labels the same neuronal types as the anti-Ilp7 antibody, thus confirming its specificity.
(C) dMP2 neurons express Ilp7 RNA, as revealed by in situ/immunofluorescence double labelling of first-instar larvae carrying a dMP2 reporter line.
(D) Ilp7 peptide sequence, with predicted signal peptide, B, C, and A chains. Optimal predicted cleavage sites, as identified in [9], are in bold. The anti-Ilp7 antibody used throughout this study was generated against the peptide sequence highlighted in green. A second anti-Ilp7 antibody, used in panels F and F', was generated against the A chain sequence highlighted in magenta.
(E–F′) Detection of mature Ilp7 B chain (E and E′) or A chain (F and F′) in or in close proximity to dMP2 terminals (green) on the visceral muscle of the hindgut (blue). See Text S1 for details.
Genotypes: (A, B) *y w*; (C) *dMP2-GAL4,UASnGFP/+*; (E–F') *odd^GAL,^,UAS-GFP/UAS-CD8-GFP*.
(1.7 MB TIF)

Figure S2 Ilp7-expressing Neurites Are in Close Proximity to Ilp2-producing mNSCs (A) Expression of nuclear GFP from an *Ilp2-GAL4* reporter line expressed in the insulin-producing mNSCs shows Ilp7-positive projections extending close to the mNSC cell bodies.
(B) Expression of a membrane-localised GFP driven by *Ilp2-Gal4* reveals apparent association between Ilp7- and Ilp2-positive neurites.
Genotypes: (A) *Ilp2-GAL4/UAS-nmEGFP; UAS-nmEGFP/+* and (B) *Ilp2-GAL4, UAS-CD8-GFP/+.*
(723 KB TIF)

Figure S3 Ilp7 RNA Expression in the Mutants Used in This Study The effect on Ilp7 expression observed for all the mutants used in this study occurs at the transcriptional level. Ilp7 RNA is reduced or absent in *wit* (B), *gbb* (D), and *Mad* (F) mutants, and reduced in *dimm* mutants (H). Each of these panels is preceded by a wild-type CNS processed in parallel to illustrate the reduction in levels (A, C, E, and G). Ilp7 is absent from *fkh* mutant dMP2s (I) and often absent from *hb9* mutant dMP2s (J). In *Abd-Bm*, *rpr*, *skl* mutants lacking Abd-B expression in A8 (arrow), no Ilp7 RNA is detected in this segment.
Genotypes: (A) *wit^A12^/+*; (B) *wit^A12^/wit^B11^*; (C) *gbb^1^/+*; (D) *gbb^1^/gbb^1^*; (E) *Mad^10^/+*; (F) *Mad^10^/Mad^12^*; (G) *dimm^P1^/+*; (H) *dimm^P1^/dimm^rev4^*; (I) *fkh^6^/fkh^6^*; (J) *hb9^KK30^/hb9^JJ154^*; and (K) *AbdB^M5^,XR38/AbdB^M5^,ED225*.
(2.2 MB TIF)

Figure S4 Ilp7 Expression in *sax* Mutants (A) Wild type expression of Ilp7 in a first-instar CNS.
(B) Ilp7 peptide is reduced or absent in dMP2 neurons of *sax* mutants.
Genotypes: (A) *y w* and (B) *sax^4^/Df(2R)NCX8*.
(790 KB TIF)

Figure S5 The Segmental Apoptosis of dMP2 Neurons Is Not Regulated by Dimm or a Retrograde BMP Signal At the end of embryogenesis, anterior dMP2 neurons have undergone apoptosis in *wit* (A), *gbb* (B), and *dimm* (C) mutants. Only posterior dMP2 neurons persist, as revealed by their expression of the dMP2 reporter (as in the wild type, the expression in A9 is weak and variable).
(D) Expression of Glued^DN^ in dMP2 and MP1 neurons with *odd^GAL4^* does not affect their segmental apoptosis. At the end of embryogenesis, dMP2 neurons are apparent in A6 to A9, whereas MP1 neurons (smaller and more medial) are present in A5 to A9.
Genotypes: (A) *UAS-CD8-GFP/+; dMP2-GAL4,wit^A12^/wit^B11^*; (B) *gbb^1^/gbb^1^*; *UAS-CD8-GFP/dMP2-GAL4*; (C) *dimm^P1^/dimm^rev4^; UAS-CD8-GFP/dMP2-GAL4*; and (D) *odd^GAL4^,UAS-GFP/UAS-Glued^DN^.*
(324 KB TIF)

Figure S6 Several Aspects of dMP2 Identity Are Not Affected in *wit* or *gbb* Mutants In *wit* mutants, dMP2 neurons express early markers in every segment, such as Odd, Fkh, and Hb9 (A–C).
Posterior dMP2 neurons express Abd-B (D).
In late embryos, posterior dMP2 neurons express Dimm (E), exit the VNC in the correct nerve (F), and innervate the hindgut (G). The same results were obtained for *gbb* mutants (H–N).
Genotypes: (A–G) *UAS-CD8-GFP/+; dMP2-GAL4,wit^A12^/wit^B11^*; (H–N) *gbb^1^/gbb^1^*; *UAS-CD8-GFP/dMP2-GAL4.*
(2.9 MB TIF)

Figure S7 The Ilp7 Code Is Specific (A) A wild-type first-instar CNS shows Ilp7 expression in three lateral pairs of TV neurons and one single pair of suboesophageal neurons.
(B) No ectopic FMRFa is observed when the Ilp7 code is misexpressed in motor neurons.
Genotypes: (A) *y w* and (B) *OK371-GAL4/UAS-Abd-B; UAS-dimm,UAS-fkh*.
(567 KB TIF)

Figure S8 Summary of *hb9*, *fkh*, and *Abd-B* Phenotypes Cartoon summarizing the dual role of Hb9 as a pro-apoptotic and pro-insulinergic factor, the effects of Fkh on all aspects of the transition from pioneer to neuroendocrine identity, and the anti-apoptotic and pro-Ilp7 functions of Abd-B. See text for details.
(1.1 MB TIF)

Text S1 Supplementary Materials and Methods (33 KB DOC)

## Abbreviations

Abd-A: Abdominal-A
Abd-B: Abdominal-B
BMP: bone morphogenetic protein
CNS: central nervous system
Dimm: Dimmed
Fkh: Fork head
FMRFa: FMRFamide
Gbb: Glass-bottom boat
GFP: green fluorescent protein
Ilp: Insulin-like peptide
Mad: Mothers against dpp
mNSCs: median neurosecretory cells
Odd: Odd-skipped
Rpr: Reaper
Sax: Saxophone
Skl: Sickle
Ubx: Ultrabithorax
VNC: ventral nerve cord
Wit: Wishful-thinking

## References

[pbio-0060058-b001] Saltiel AR, Kahn CR (2001). Insulin signalling and the regulation of glucose and lipid metabolism. Nature.

[pbio-0060058-b002] Biddinger SB, Kahn CR (2006). From mice to men: insights into the insulin resistance syndromes. Annu Rev Physiol.

[pbio-0060058-b003] Marshall S (2006). Role of insulin, adipocyte hormones, and nutrient-sensing pathways in regulating fuel metabolism and energy homeostasis: a nutritional perspective of diabetes, obesity, and cancer. Sci STKE.

[pbio-0060058-b004] Murtaugh LC (2007). Pancreas and beta-cell development: from the actual to the possible. Development.

[pbio-0060058-b005] Murtaugh LC, Melton DA (2003). Genes, signals, and lineages in pancreas development. Annu Rev Cell Dev Biol.

[pbio-0060058-b006] Wilson ME, Scheel D, German MS (2003). Gene expression cascades in pancreatic development. Mech Dev.

[pbio-0060058-b007] Nassel DR (2002). Neuropeptides in the nervous system of Drosophila and other insects: multiple roles as neuromodulators and neurohormones. Prog Neurobiol.

[pbio-0060058-b008] Wu Q, Brown MR (2006). Signaling and function of insulin-like peptides in insects. Annu Rev Entomol.

[pbio-0060058-b009] Brogiolo W, Stocker H, Ikeya T, Rintelen F, Fernandez R (2001). An evolutionarily conserved function of the Drosophila insulin receptor and insulin-like peptides in growth control. Curr Biol.

[pbio-0060058-b010] Wang S, Tulina N, Carlin DL, Rulifson EJ (2007). The origin of islet-like cells in Drosophila identifies parallels to the vertebrate endocrine axis. Proc Natl Acad Sci U S A.

[pbio-0060058-b011] Bateman JM, McNeill H (2004). Temporal control of differentiation by the insulin receptor/tor pathway in Drosophila. Cell.

[pbio-0060058-b012] Bohni R, Riesgo-Escovar J, Oldham S, Brogiolo W, Stocker H (1999). Autonomous control of cell and organ size by CHICO, a Drosophila homolog of vertebrate IRS1–4. Cell.

[pbio-0060058-b013] Broughton SJ, Piper MD, Ikeya T, Bass TM, Jacobson J (2005). Longer lifespan, altered metabolism, and stress resistance in Drosophila from ablation of cells making insulin-like ligands. Proc Natl Acad Sci U S A.

[pbio-0060058-b014] Fernandez R, Tabarini D, Azpiazu N, Frasch M, Schlessinger J (1995). The Drosophila insulin receptor homolog: a gene essential for embryonic development encodes two receptor isoforms with different signaling potential. Embo J.

[pbio-0060058-b015] Ikeya T, Galic M, Belawat P, Nairz K, Hafen E (2002). Nutrient-dependent expression of insulin-like peptides from neuroendocrine cells in the CNS contributes to growth regulation in Drosophila. Curr Biol.

[pbio-0060058-b016] Rulifson EJ, Kim SK, Nusse R (2002). Ablation of insulin-producing neurons in flies: growth and diabetic phenotypes. Science.

[pbio-0060058-b017] Arendt D, Nubler-Jung K (1996). Common ground plans in early brain development in mice and flies. Bioessays.

[pbio-0060058-b018] Bate CM (1976). Pioneer neurones in an insect embryo. Nature.

[pbio-0060058-b019] Bentley D, Keshishian H (1982). Pathfinding by peripheral pioneer neurons in grasshoppers. Science.

[pbio-0060058-b020] Arendt D, Nubler-Jung K (1999). Comparison of early nerve cord development in insects and vertebrates. Development.

[pbio-0060058-b021] Goodman CS, Doe CQ, Bate M, Martinez-Arias A (1993). Embryonic development of the Drosophila central nervous system. The development of Drosophila melanogaster.

[pbio-0060058-b022] Raper JA, Tessier-Lavigne M, Bloom FE, Landis SE, Roberts JL, Squire LR, Zigmond MJ (1998). Growth cones and axon pathfinding. Fundamental neuroscience.

[pbio-0060058-b023] Hidalgo A, Brand AH (1997). Targeted neuronal ablation: the role of pioneer neurons in guidance and fasciculation in the CNS of Drosophila. Development.

[pbio-0060058-b024] Klose M, Bentley D (1989). Transient pioneer neurons are essential for formation of an embryonic peripheral nerve. Science.

[pbio-0060058-b025] Raper JA, Bastiani MJ, Goodman CS (1984). Pathfinding by neuronal growth cones in grasshopper embryos. IV. The effects of ablating the A and P axons upon the behavior of the G growth cone. J Neurosci.

[pbio-0060058-b026] Kutsch W, Bentley D (1987). Programmed death of peripheral pioneer neurons in the grasshopper embryo. Dev Biol.

[pbio-0060058-b027] Truman JW (1984). Cell death in invertebrate nervous systems. Annu Rev Neurosci.

[pbio-0060058-b028] Miguel-Aliaga I, Thor S (2004). Segment-specific prevention of pioneer neuron apoptosis by cell-autonomous, postmitotic Hox gene activity. Development.

[pbio-0060058-b029] Doe CQ, Hiromi Y, Gehring WJ, Goodman CS (1988). Expression and function of the segmentation gene fushi tarazu during Drosophila neurogenesis. Science.

[pbio-0060058-b030] Spana EP, Doe CQ (1996). Numb antagonizes Notch signaling to specify sibling neuron cell fates. Neuron.

[pbio-0060058-b031] Spana EP, Kopczynski C, Goodman CS, Doe CQ (1995). Asymmetric localization of numb autonomously determines sibling neuron identity in the Drosophila CNS. Development.

[pbio-0060058-b032] Thomas JB, Bastiani MJ, Bate M, Goodman CS (1984). From grasshopper to Drosophila: a common plan for neuronal development. Nature.

[pbio-0060058-b033] Larsen C, Franch-Marro X, Hartenstein V, Alexandre C, Vincent JP (2006). An efficient promoter trap for detection of patterned gene expression and subsequent functional analysis in Drosophila. Proc Natl Acad Sci U S A.

[pbio-0060058-b034] Allan DW, St Pierre SE, Miguel-Aliaga I, Thor S (2003). Specification of neuropeptide cell identity by the integration of retrograde BMP signaling and a combinatorial transcription factor code. Cell.

[pbio-0060058-b035] Marques G, Bao H, Haerry TE, Shimell MJ, Duchek P (2002). The Drosophila BMP type II receptor Wishful Thinking regulates neuromuscular synapse morphology and function. Neuron.

[pbio-0060058-b036] Hewes RS, Park D, Gauthier SA, Schaefer AM, Taghert PH (2003). The bHLH protein Dimmed controls neuroendocrine cell differentiation in Drosophila. Development.

[pbio-0060058-b037] Miguel-Aliaga I, Allan DW, Thor S (2004). Independent roles of the dachshund and eyes absent genes in BMP signaling, axon pathfinding and neuronal specification. Development.

[pbio-0060058-b038] Broihier HT, Skeath JB (2002). Drosophila homeodomain protein dHb9 directs neuronal fate via crossrepressive and cell-nonautonomous mechanisms. Neuron.

[pbio-0060058-b039] Odden JP, Holbrook S, Doe CQ (2002). Drosophila HB9 is expressed in a subset of motoneurons and interneurons, where it regulates gene expression and axon pathfinding. J Neurosci.

[pbio-0060058-b040] Rogulja-Ortmann A, Luer K, Seibert J, Rickert C, Technau GM (2007). Programmed cell death in the embryonic central nervous system of Drosophila melanogaster. Development.

[pbio-0060058-b041] Mann RS, Morata G (2000). The developmental and molecular biology of genes that subdivide the body of Drosophila. Annu Rev Cell Dev Biol.

[pbio-0060058-b042] McGinnis W, Krumlauf R (1992). Homeobox genes and axial patterning. Cell.

[pbio-0060058-b043] Casanova J, White RA (1987). Trans-regulatory functions in the Abdominal-B gene of the bithorax complex. Development.

[pbio-0060058-b044] Mahr A, Aberle H (2006). The expression pattern of the Drosophila vesicular glutamate transporter: a marker protein for motoneurons and glutamatergic centers in the brain. Gene Expression Patterns.

[pbio-0060058-b045] Goodman CS, Bate M, Spitzer NC (1981). Embryonic development of identified neurons: origin and transformation of the H cell. J Neurosci.

[pbio-0060058-b046] Budnik V, Wu CF, White K (1989). Altered branching of serotonin-containing neurons in Drosophila mutants unable to synthesize serotonin and dopamine. J Neurosci.

[pbio-0060058-b047] Wheeler SR, Kearney JB, Guardiola AR, Crews ST (2006). Single-cell mapping of neural and glial gene expression in the developing Drosophila CNS midline cells. Dev Biol.

[pbio-0060058-b048] Hartenstein V (2006). The neuroendocrine system of invertebrates: a developmental and evolutionary perspective. J Endocrinol.

[pbio-0060058-b049] Ghysen A (2003). The origin and evolution of the nervous system. Int J Dev Biol.

[pbio-0060058-b050] Arber S, Han B, Mendelsohn M, Smith M, Jessell TM (1999). Requirement for the homeobox gene Hb9 in the consolidation of motor neuron identity. Neuron.

[pbio-0060058-b051] Thaler J, Harrison K, Sharma K, Lettieri K, Kehrl J (1999). Active suppression of interneuron programs within developing motor neurons revealed by analysis of homeodomain factor HB9. Neuron.

[pbio-0060058-b052] Friedman JR, Kaestner KH (2006). The Foxa family of transcription factors in development and metabolism. Cell Mol Life Sci.

[pbio-0060058-b053] Salio C, Lossi L, Ferrini F, Merighi A (2006). Neuropeptides as synaptic transmitters. Cell Tissue Res.

[pbio-0060058-b054] Strand FL (1999). Neuropeptides, regulators of physiological processes.

[pbio-0060058-b055] Ferri AL, Lin W, Mavromatakis YE, Wang JC, Sasaki H (2007). Foxa1 and Foxa2 regulate multiple phases of midbrain dopaminergic neuron development in a dosage-dependent manner. Development.

[pbio-0060058-b056] Jacob J, Ferri AL, Milton C, Prin F, Pla P (2007). Transcriptional repression coordinates the temporal switch from motor to serotonergic neurogenesis. Nat Neurosci.

[pbio-0060058-b057] Norton WH, Mangoli M, Lele Z, Pogoda HM, Diamond B (2005). Monorail/Foxa2 regulates floorplate differentiation and specification of oligodendrocytes, serotonergic raphe neurones and cranial motoneurons. Development.

[pbio-0060058-b058] Bossing T, Technau GM (1994). The fate of the CNS midline progenitors in Drosophila as revealed by a new method for single cell labelling. Development.

[pbio-0060058-b059] Mizusawa N, Hasegawa T, Ohigashi I, Tanaka-Kosugi C, Harada N (2004). Differentiation phenotypes of pancreatic islet beta- and alpha-cells are closely related with homeotic genes and a group of differentially expressed genes. Gene.

[pbio-0060058-b060] Johnson JD, Ahmed NT, Luciani DS, Han Z, Tran H (2003). Increased islet apoptosis in Pdx1+/− mice. J Clin Invest.

[pbio-0060058-b061] Kuzin A, Brody T, Moore AW, Odenwald WF (2005). Nerfin-1 is required for early axon guidance decisions in the developing Drosophila CNS. Dev Biol.

[pbio-0060058-b062] Gierl MS, Karoulias N, Wende H, Strehle M, Birchmeier C (2006). The zinc-finger factor Insm1 (IA-1) is essential for the development of pancreatic beta cells and intestinal endocrine cells. Genes Dev.

[pbio-0060058-b063] Gao N, White P, Doliba N, Golson ML, Matschinsky FM (2007). Foxa2 controls vesicle docking and insulin secretion in mature Beta cells. Cell Metab.

[pbio-0060058-b064] Sund NJ, Vatamaniuk MZ, Casey M, Ang SL, Magnuson MA (2001). Tissue-specific deletion of Foxa2 in pancreatic beta cells results in hyperinsulinemic hypoglycemia. Genes Dev.

[pbio-0060058-b065] Sussel L, Kalamaras J, Hartigan-O'Connor DJ, Meneses JJ, Pedersen RA (1998). Mice lacking the homeodomain transcription factor Nkx2.2 have diabetes due to arrested differentiation of pancreatic beta cells. Development.

[pbio-0060058-b066] McDonald JA, Holbrook S, Isshiki T, Weiss J, Doe CQ (1998). Dorsoventral patterning in the Drosophila central nervous system: the vnd homeobox gene specifies ventral column identity. Genes Dev.

[pbio-0060058-b067] Broihier HT, Kuzin A, Zhu Y, Odenwald W, Skeath JB (2004). Drosophila homeodomain protein Nkx6 coordinates motoneuron subtype identity and axonogenesis. Development.

[pbio-0060058-b068] Goulley J, Dahl U, Baeza N, Mishina Y, Edlund H (2007). BMP4-BMPR1A signaling in beta cells is required for and augments glucose-stimulated insulin secretion. Cell Metab.

[pbio-0060058-b069] Harrison KA, Thaler J, Pfaff SL, Gu H, Kehrl JH (1999). Pancreas dorsal lobe agenesis and abnormal islets of Langerhans in Hlxb9-deficient mice. Nat Genet.

[pbio-0060058-b070] Li H, Arber S, Jessell TM, Edlund H (1999). Selective agenesis of the dorsal pancreas in mice lacking homeobox gene Hlxb9. Nat Genet.

[pbio-0060058-b071] Weigel D, Jurgens G, Kuttner F, Seifert E, Jackle H (1989). The homeotic gene fork head encodes a nuclear protein and is expressed in the terminal regions of the Drosophila embryo. Cell.

[pbio-0060058-b072] Sekelsky JJ, Newfeld SJ, Raftery LA, Chartoff EH, Gelbart WM (1995). Genetic characterization and cloning of mothers against dpp, a gene required for decapentaplegic function in Drosophila melanogaster. Genetics.

[pbio-0060058-b073] Bossing T, Brand AH (2002). Dephrin, a transmembrane ephrin with a unique structure, prevents interneuronal axons from exiting the Drosophila embryonic CNS. Development.

[pbio-0060058-b074] Kusch T, Reuter R (1999). Functions for Drosophila brachyenteron and forkhead in mesoderm specification and cell signalling. Development.

[pbio-0060058-b075] Lee T, Luo L (2001). Mosaic analysis with a repressible cell marker (MARCM) for Drosophila neural development. Trends Neurosci.

[pbio-0060058-b076] Zhou L, Schnitzler A, Agapite J, Schwartz LM, Steller H (1997). Cooperative functions of the reaper and head involution defective genes in the programmed cell death of Drosophila central nervous system midline cells. Proc Natl Acad Sci U S A.

[pbio-0060058-b077] Kosman D, Small S, Reinitz J (1998). Rapid preparation of a panel of polyclonal antibodies to Drosophila segmentation proteins. Dev Genes Evol.

[pbio-0060058-b078] Allan DW, Park D, St Pierre SE, Taghert PH, Thor S (2005). Regulators acting in combinatorial codes also act independently in single differentiating neurons. Neuron.

[pbio-0060058-b079] Tanimoto H, Itoh S, ten Dijke P, Tabata T (2000). Hedgehog creates a gradient of DPP activity in Drosophila wing imaginal discs. Mol Cell.

[pbio-0060058-b080] White RA, Wilcox M (1984). Protein products of the bithorax complex in Drosophila. Cell.

[pbio-0060058-b081] Macias A, Casanova J, Morata G (1990). Expression and regulation of the abd-A gene of Drosophila. Development.

