# Supplementary material for: Postmitotic Specification of Drosophila Insulinergic Neurons from Pioneer Neurons
Source: PLoS Biol. 2008 Mar 11;6(3):e58. doi: 10.1371/journal.pbio.0060058 (PMC2265769; doi:10.1371/journal.pbio.0060058)
Supplement: Supplementary file 1 [file pbio.0060058.sd001.doc]

**Supplemental Materials and Methods**

Generation of an antibody to the A-chain of Ilp7

In addition to the antibody directed against the B chain of Ilp7, used throughout this study (see Materials and Methods for details), a second α-Ilp7 rabbit antibody was generated against the synthetic peptide SDGNTPSISNECCTKAGCTW. This sequence is within the predicted A chain, and corresponds to amino acids 124-143 of the predicted Ilp7 protein. Peptide synthesis, antibody generation and affinity purification were done by Eurogentec. Ilp7 immunoreactivity was found to be identical to that observed with the other α-Ilp7 antibody and to Ilp7 RNA expression. This antibody was used at a 1:5000 dilution.

Detection of extracellular Ilp7 peptide

To establish whether the Ilp7 antibody can detect mature Ilp7 peptide, we adapted the protocol routinely used to detect the extracellular fraction of signalling molecules such as Dpp and Wg [1,2] to detection of mature Ilp7 peptide on the hindgut. Third-instar larval hindguts were incubated them at room temperature with antibody against the A or B chain of Ilp7 diluted 1:1500 in PBS with 4% normal goat serum for 40 minutes. Hindguts when then fixed in 4%PFA for 20 minutes and subsequently processed as for standard total immunostains. Incubation of intact, non-permeabilized cells with the primary antibody prior to fixation only detects extracellular Ilp7, or Ilp7 peptide that has been endocytosed following its initial secretion or expression on the cell surface. With this method, we detected mature peptide in two out of five hindguts with the B chain antibody, and in two out of eight hindguts with the A chain antibody.

*In situ* detection of Ilp7 mRNA

CNSs were dissected and fixed on polyLysine-coated slides. In situ hybridisation was carried out as previously described [3].

**Supplemental References**

1. Belenkaya TY, Han C, Yan D, Opoka RJ, Khodoun M, et al. (2004) Drosophila Dpp morphogen movement is independent of dynamin-mediated endocytosis but regulated by the glypican members of heparan sulfate proteoglycans. Cell 119: 231-244.

2. Strigini M, Cohen SM (2000) Wingless gradient formation in the Drosophila wing. Curr Biol 10: 293-300.

3. Tautz D, Pfeifle C (1989) A non-radioactive in situ hybridization method for the localization of specific RNAs in Drosophila embryos reveals translational control of the segmentation gene hunchback. Chromosoma 98: 81-85.
